# What Mechanisms Are Responsible for the Reuptake of Levodopa-Derived Dopamine in Parkinsonian Striatum?

**DOI:** 10.3389/fnins.2016.00575

**Published:** 2016-12-15

**Authors:** Haruo Nishijima, Masahiko Tomiyama

**Affiliations:** ^1^Department of Neurology, Aomori Prefectural Central HospitalAomori, Japan; ^2^Department of Neurophysiology, Institute of Brain Science, Hirosaki University Graduate School of MedicineHirosaki, Japan

**Keywords:** dopamine, levodopa, norepinephrine, Parkinson's disease, striatum, serotonin, transporter

## Abstract

Levodopa is the most effective medication for motor symptoms in Parkinson's disease. However, various motor and non-motor complications are associated with levodopa treatment, resulting from altered levodopa-dopamine metabolism with disease progression and long-term use of the drug. The present review emphasizes the role of monoamine transporters other than the dopamine transporter in uptake of extracellular dopamine in the dopamine-denervated striatum. When dopaminergic neurons are lost and dopamine transporters decreased, serotonin and norepinephrine transporters compensate by increasing uptake of excessive extracellular dopamine in the striatum. Organic cation transporter-3 and plasma membrane monoamine transporter, low affinity, and high capacity transporters, also potentially uptake dopamine when high-affinity transporters do not work normally. Selective serotonin reuptake inhibitors and serotonin norepinephrine reuptake inhibitors are often administered to patients with Parkinson's disease presenting with depression, pain or other non-motor symptoms. Thus, it is important to address the potential of these drugs to modify dopamine metabolism and uptake through blockade of the compensatory function of these transporters, which could lead to changes in motor symptoms of Parkinson's disease.

## Introduction

Parkinson's disease (PD) is a neurodegenerative disease associated with progressive loss of nigrostriatal dopaminergic neurons. A patient with PD presents motor symptoms such as tremor, akinesia, hypokinesia, rigidity, and postural disturbance, and non-motor symptoms such as pain, sleep disturbance, apathy, depression, and constipation (Jankovic, 2008). Levodopa is the most effective drug to alleviate motor symptoms of PD (Mercuri and Bernardi, 2005; Smith et al., 2012); however, levodopa-induced motor and non-motor complications occur with long-term use of the drug (Aquino and Fox, 2015; Beaulieu-Boire and Lang, 2015). Levodopa-induced complications are mainly due to altered levodopa-dopamine metabolism in the brain and the narrowing of the therapeutic window in patients with PD (Olanow and Obeso, 2000; Pavese et al., 2006). Striatal function targeted by levodopa is deeply involved with levodopa-associated motor complications such as wearing-off and levodopa-induced dyskinesia (de la Fuente-Fernández et al., 2004). Detailed mechanisms of levodopa-dopamine metabolism in the striatum of patients with PD are far from evident yet, however, a number of studies have shed light on this issue using animal models of PD and human subjects.

**Figure 1:**
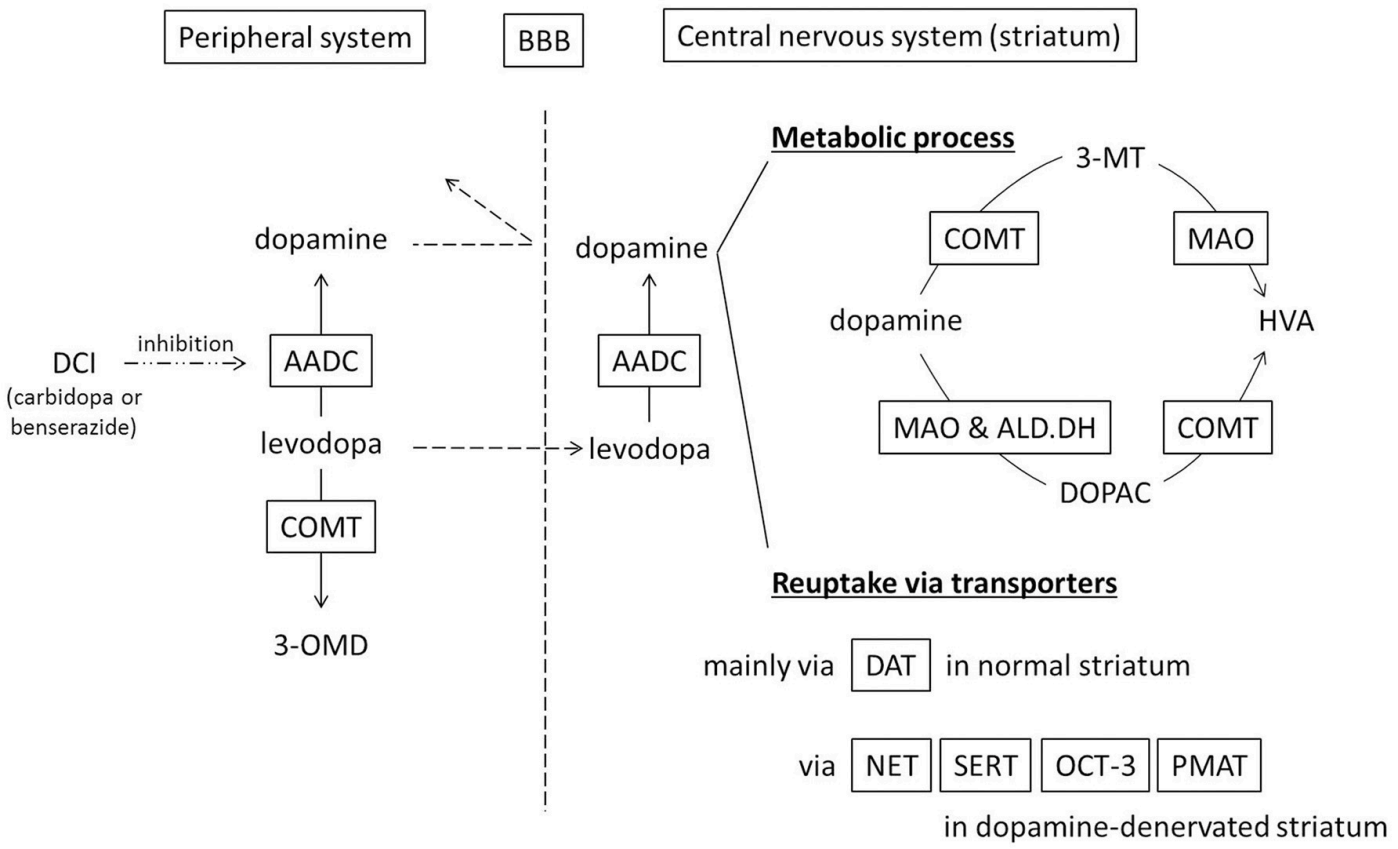
**Schematic diagram showing metabolism and uptake of levodopa-derived dopamine**. Levodopa therapy is usually combined with a dopa-decarboxylase inhibitor (carbidopa or benserazide) in order to minimize peripheral conversion of levodopa. 3-MT, 3-methoxytyramine; 3-OMD, 3-O-methyldopa; AADC, aromatic amino acid decarboxylase; ALD.DH, aldehyde dehydrogenase; BBB, blood–brain barrier; COMT, catechol-O-methyltransferase; DAT, dopamine transporter; DCI, dopa-decarboxylase inhibitor; DOPAC, dihydroxyphenylacetic acid; HVA, homovanillic acid; MAO, monoamine oxidase; NET, norepinephrine transporter, noradrenaline transporter; OCT-3, organic cation transporter-3; PMAT, plasma membrane monoamine transporter; SERT, serotonin transporter.

**Figure 2:**
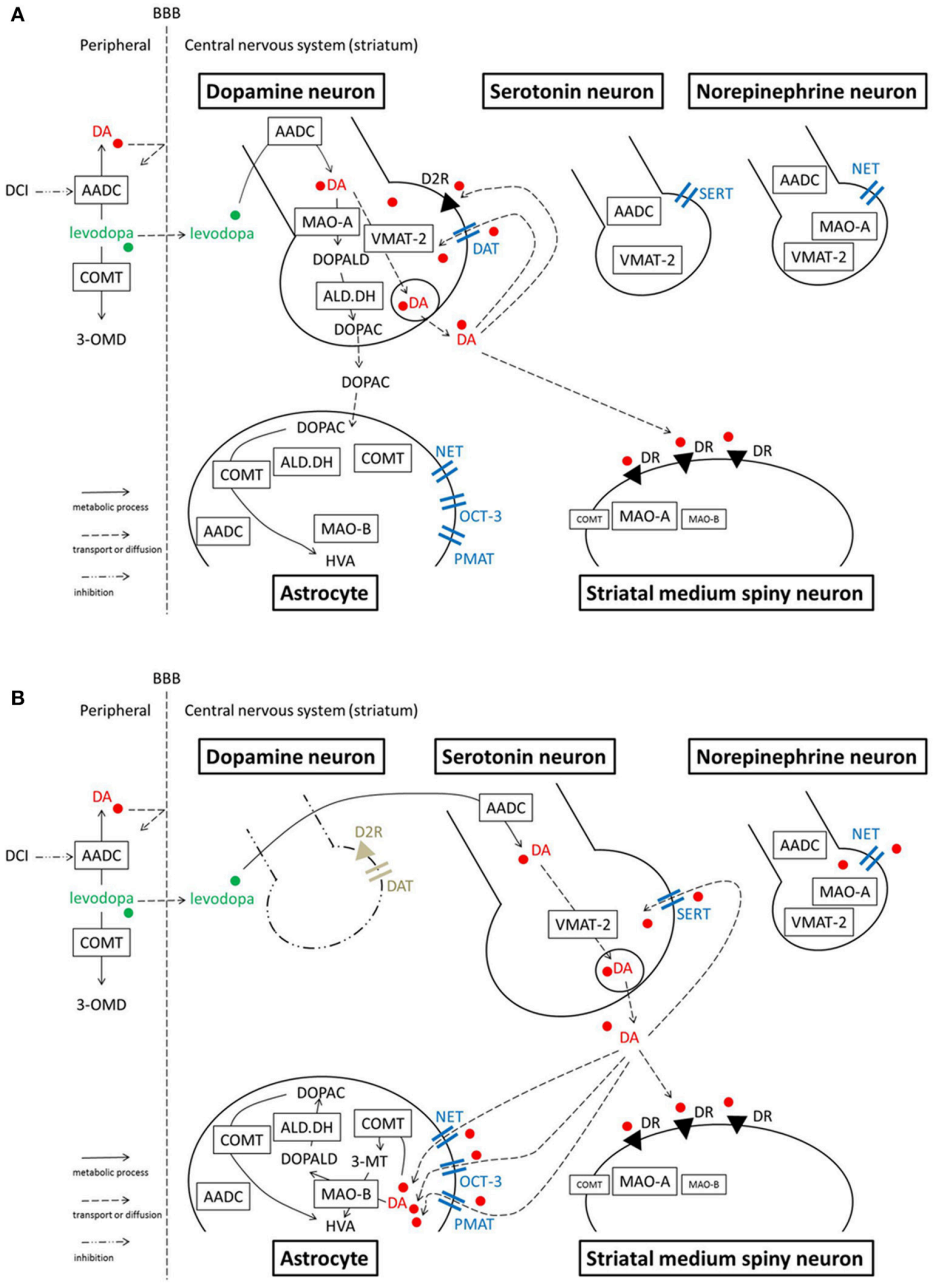
**Model illustrating the processes of levodopa-dopamine metabolism and uptake. (A)** Normal striatum **(B)** Dopamine-denervated striatum. Note that MAO-A localization is unclear. A study suggests MAO-A is mainly expressed in medium spiny neurons, rather than in axon terminals in the striatum. Serotonergic neurons express MAO-B in their cell bodies, however, it is still unclear whether the neurons express MAO in axon terminals. OCT-3 also exists in neurons, however the specific subtype is unknown. PMAT is illustrated at the astrocyte membrane according to one recent study (see text), although its localization is not certain yet. 3-MT, 3-methoxytyramine; 3-OMD, 3-O-methyldopa; AADC, aromatic amino acid decarboxylase; ALD.DH, aldehyde dehydrogenase; COMT, catechol-O-methyltransferase; D2R, dopamine D2 receptor; DA, dopamine; DAT, dopamine transporter; DCI, dopa-decarboxylase inhibitor; DOPAC, dihydroxyphenylacetic acid; DOPALD, dihydroxyphenylacetaldehyde; DR, dopamine receptor; HVA, homovanillic acid; MAO, monoamine oxidase; NET, norepinephrine transporter, noradrenaline transporter; OCT, organic cation transporter; PMAT, plasma membrane monoamine transporter; SERT, serotonin transporter; VMAT-2, vesicular monoamine transporter-2.

In the peripheral system (Figure 1), levodopa administered systemically, whether oral or intravenous, undergoes decarboxylation by aromatic amino acid decarboxylase (AADC) and is converted to dopamine. Peripheral dopamine cannot cross the blood-brain barrier and enter the brain (Nutt et al., 1985; Cedarbaum, 1987). Some administered levodopa is O-methylated by catechol-O-methyltransferase (COMT) and converted to 3-O-methyldopa (3-OMD), which does not work as a dopaminergic neurotransmitter in the striatum (Kaakkola, 2000). The remaining levodopa can cross the blood-brain barrier and reach the central nervous system. In the normal striatum, levodopa is metabolized, released, and reuptaken mainly by dopaminergic neurons. Dopaminergic neurons convert levodopa into dopamine by AADC, and store dopamine in the synaptic vesicles by vesicular monoamine transporter-2 (VMAT-2; Wimalasena, 2011), and release dopamine to the synaptic cleft. Dopamine transporters (DAT) are expressed at terminals of dopaminergic neurons (Nirenberg et al., 1996) and reuptake extracellular dopamine for reuse or metabolization (Cass et al., 1993; Cass and Gerhardt, 1994; Giros et al., 1996; Jaber et al., 1997; Mundorf et al., 2001; Rice et al., 2011). With DAT and negative feedback by D2 dopamine receptors (Rice et al., 2011), dopaminergic neurons can control the concentration of extracellular dopamine levels (Figure 2A).

When dopaminergic neurons projecting to the striatum are markedly decreased in PD, serotonergic neurons play a significant role in converting exogenous levodopa into dopamine, storing dopamine, and releasing dopamine into synapses in the striatum (Ng et al., 1970, 1971; Hollister et al., 1979; Arai et al., 1994, 1995, 1996; Tanaka et al., 1999; Kannari et al., 2001; Maeda et al., 2003, 2005; Politis et al., 2010; Cheshire and Williams, 2012; Politis et al., 2014; Figure 2B). However, serotonergic neurons do not have dopamine D2 receptors; the absence of negative feedback allows uncontrolled synthesis and release of dopamine from serotonergic neurons. Furthermore, because serotonergic neurons do not express DAT, they cannot reuptake dopamine from the synaptic cleft efficiently. Non-regulated release of dopamine induces pulsatile fluctuations of extracellular dopamine concentrations in the striatum, leading to motor complications in advanced PD patients such as levodopa-induced dyskinesia (Olanow and Obeso, 2000; Pavese et al., 2006; Cheshire and Williams, 2012; Politis et al., 2014).

Dopamine in the striatum is metabolized by monoamine oxidase (MAO)-A, MAO-B, aldehyde dehydrogenase (ALD.DH), and COMT into homovanillic acid (HVA; Kaakkola, 2000; Gesi et al., 2001; Figure 1). In the striatum, MAO-A exists at the outer membrane of mitochondria in the axon terminals of catecholamine neurons (Westlund et al., 1993) or in medium spiny neurons (Sader-Mazbar et al., 2013). The majority of MAO-B is detected in astrocytes in the brain (Levitt et al., 1982; Westlund et al., 1985; Ekblom et al., 1993; Sader-Mazbar et al., 2013). Some MAO-B activity is detected in medium spiny neurons (Sader-Mazbar et al., 2013) and perikarya of serotonergic neurons (Levitt et al., 1982; Arai et al., 1998, 2002). COMT is primarily expressed in glial cells, and some in postsynaptic cells (Männistö and Kaakkola, 1999; Kaakkola, 2000). Soluble COMT exists in striatal glial cells, whereas membrane-bound COMT exists in postsynaptic neurons and non-neuronal cells (Kaakkola et al., 1987). Dopamine is metabolized into dihydroxyphenylacetaldehyde (DOPALD) via MAO-A or MAO-B. DOPALD is converted to dihydroxyphenylacetic acid (DOPAC) by ALD.DH, which is finally metabolized into HVA by COMT. COMT in the central nervous system converts dopamine into 3-methoxytyramine (3-MT), which is then metabolized into HVA via MAO (Männistö and Kaakkola, 1999; Kaakkola, 2000; Figures 1, 2A,B).

Extracellular dopamine is reuptaken into neurons or glial cells to be metabolized by MAO and COMT as described above, or to be reused. Despite a >70% striatal DAT loss in a PD model rat, dopamine uptake decreases only 25%, and dopamine uptake per remaining DAT protein increases as DAT loss approaches 99% (Chotibut et al., 2012). This can be explained by high affinity of DAT for dopamine and enough capacity to transport available dopamine in the striatum, however, an alternate explanation is that transporters other than DAT compensate for DAT loss. Recent studies have demonstrated that serotonin transporters (SERT) and norepinephrine transporters (NET) have a greater role in reuptake of extracellular dopamine in the highly dopamine-denervated striatum where DAT is also lost (Chotibut et al., 2012). Thus, it is probable that selective serotonin reuptake inhibitors (SSRI) and serotonin and norepinephrine reuptake inhibitors (SNRI), which are often administered to PD patients in clinical practice, can influence the motor effects of levodopa.

The aim of this review is to summarize recent advances in understanding the role of high-affinity transporters, SERT, and NET, in reuptake of extracellular dopamine in the striatum. It will also address low affinity and high capacity transporters, organic cation transporter-3 (OCT-3), and plasma membrane monoamine transporter (PMAT) in the striatum, which can also reuptake extracellular dopamine and are gaining increasing attention.

## The role of serotonin transporters in reuptake of dopamine in the dopamine-denervated striatum

SERT has a high affinity for serotonin uptake and the majority of these transporters exist on the axonal plasma membranes of serotonin fibers (Zhou et al., 1998). SERT can also transport norepinephrine and dopamine, particularly at high dopamine concentrations (Vizi et al., 2004; Larsen et al., 2011). In a parkinsonian model rat brain in which DAT is absent, levodopa-derived excessive dopamine can be taken up by serotonergic neurons via SERT (Kannari et al., 2006). In a study using a 6-hydroxydopamine (OHDA)-lesioned hemiparkinsonian rat and microdialysis tecnique (Kannari et al., 2006), acute local perfusion of the SSRI fluoxetine in the striatum increases levodopa-derived extracellular dopamine levels. Conversely, the same research group demonstrated that acute systemic administration of fluoxetine decreases levodopa-derived extracellular dopamine levels in the striatum of the same rat model (Yamato et al., 2001). They discussed that systemic administration of fluoxetine mainly increases extracellular serotonin at the dorsal raphe nucleus, activating 5-HT 1A autoreceptors; this results in reduced activity of serotonergic neurons, including those sending efferent fibers to the striatum, and reduced release of levodopa-derived dopamine from the serotonergic nerve terminals (Yamato et al., 2001).

Bishop et al. demonstrated that acute systemic administration of SSRIs, paroxetine, citalopram or fluoxetine, to 6-OHDA-lesioned hemiparkinsonian rats attenuates dyskinesia-like behavior without reducing the anti-parkinsonian effect of levodopa (Bishop et al., 2012). In this study, SSRIs had no effect on apomorphine-induced abnormal involuntary movements, suggesting that SSRIs modify the effects of levodopa-derived dopamine. They also examined tissue dopamine content in the dopamine-denervated striatum after levodopa and citalopram treatment, showing no significant changes compared with levodopa treatment. Effects of acute systemic SSRI treatment on striatal levodopa-derived dopamine levels are different between the study of Yamato et al. (decrease) and that of Bishop et al. (no significant change). Yamato et al. (2001) examined effects of SSRI on striatal extracellular dopamine after levodopa treatment using microdialysis, which means that the changes should reflect alteration of synaptic dopamine level. On the other hand Bishop et al. (2012) measured tissue dopamine content, meaning that they examined all synaptic, and stored dopamine. The difference in determined dopamine may account for the difference between the two reports. This discrepancy might be also due to their 6-OHDA lesioning for the rat PD model. Yamato et al. confirmed dopamine-denervation with a behavioral test using apomorphine, indicating that their model rats lost over 99% of striatal dopaminergic neurons (Tanaka et al., 1999; Yamato et al., 2001). On the other hand, Bishop et al. did not show any validation for the extent of dopamine-denervation after 6-OHDA lesioning in their paper (Bishop et al., 2012). Residual dopaminergic neurons and endogenous dopamine release may account for relatively low impact of acute SSRI treatment on striatal levodopa-derived dopamine content in their study. In the other study Bishop and his colleagues have shown that repetitive chronic systemic administration of SSRIs (3 weeks) increased striatal dopamine concentration after levodopa treatment in the same rat PD model (Conti et al., 2014). In this study, prolonged SSRI treatment attenuated dyskinesia expression and development in levodopa-primed and -naïve animals, respectively, without interfering with motor performance. They discussed that this anti-dyskinetic effect of SSRI is provided in part by 5-HT 1A receptors, while maintaining levodopa's anti-parkinsonian efficacy by enhancing striatal dopamine levels. In other words, it can be speculated that the chronic SSRI treatment in this study reduced dopamine release from serotoninergic neurons and reduced the uptake of extracellular dopamine via SERT, resulting in reduced fluctuation of extracellular dopamine concentration in the striatum. This might lead to relatively continuous dopaminergic stimulation rather than pulsatile, attenuating levodopa-induced dyskinesia although the tissue dopamine level increased in the striatum after chronic SSRI treatment. Thus, these studies using rodent PD model indicate that serotonergic neurons not only release levodopa-derived dopamine but also reuptake extracellular dopamine via SERT in parkinsonian striatum (Figure 2B).

Clinical studies of the motor effects of SSRI treatment in PD patients have yielded conflicting results; SSRIs have been shown to improve (Rampello et al., 2002), worsen (Linazasoro, 2000), or have no influence (Chung et al., 2005) over the anti-parkinsonian efficacy of levodopa. As described above, SSRIs potentially increase dopamine concentration in the striatum via SERT blockade, although they may attenuate activity of serotonergic neurons and reduce levodopa-derived dopamine release (Yamato et al., 2001). Thus, to date no SSRI is clinically applied to treat motor symptoms of PD. The clinical use of SSRIs and other monoamine transporter inhibitors for PD have been recently discussed in a comprehensive review by Huot et al. (2015). In clinical practice, major clinical effects of SSRIs on PD motor symptoms are not seen so frequently. Most of preclinical data is acute administration of SSRI rather than chronic, with supra-optimal levodopa doses that may be therapeutically irrelevant. These may explain the discrepancy between experimental data and clinical situations.

## The role of norepinephrine transporters in reuptake of dopamine in the dopamine-denervated striatum

NET has affinity not only for norepinephrine but also for dopamine (Horn, 1973; Raiteri et al., 1977). The transporter's affinity for dopamine is as strong as that of DAT (Gu et al., 1994; Giros et al., 1996; Eshleman et al., 1999). While reuptake of dopamine occurs mainly via DAT in the normal striatum (Moron et al., 2002), in brain regions with sparse dopamine innervations, such as the prefrontal cortex, NET is a major site for dopamine uptake (Moron et al., 2002).

In normal striatum, norepinephrinergic neurons and NET are sparsely expressed. However, studies suggest NET plays a larger role in the dopamine-denervated striatum. NET expression is increased and NET reuptakes extracellular dopamine in the 6-OHDA-lesioned parkinsonian striatum of a rat (Chotibut et al., 2012). Acute systemic administration of desipramine (25 mg/kg), a selective NET inhibitor, increases the extracellular dopamine concentration after levodopa treatment in the striatum of this PD model (Arai et al., 2008). Chronic administration of desipramine (12 mg/kg) also increases the striatal dopamine concentration and enhances dyskinesia-like behaviors induced by levodopa injection to this model rat (Chotibut et al., 2014). These studies suggest that NET has a significant role in reuptake of extracellular dopamine in the PD striatum. However, the targeted NETs are not necessarily expressed by norepinephrinergic neurons. In the striatum of a 6-OHDA-lesioned hemiparkinsonian rat, NET expression increases but norepinephrine tissue content remains the same (Chotibut et al., 2012). Navailles et al. (2014) showed in a similar rat model that destruction of norepinephrine fibers using N-(2-chloroethyl)-N-ethyl-2-bromobenzylamine does not potentiate the levodopa effect in the striatum. These results suggest that increased NET is expressed on cells other than norepinephrinergic neurons. The increased expression could occur on glial cells, which also express NETs (Takeda et al., 2002; Inazu et al., 2003). In cultured astrocytes from rat cortex, a NET blocker inhibits uptake of dopamine and norepinephrine; the effect was not seen with a DAT blocker, indicating dopamine uptake is performed by NETs in astrocytes (Takeda et al., 2002). In a parkinsonian state, the number of astrocytes increase with dopaminergic denervation (Teismann and Schulz, 2004; Asanuma et al., 2014). Thus, increased NET activity of dopamine transport in the dopamine-denervated striatum may occur at astrocytes, not at norepinephrinergic neurons (Figure 2B). Another possible source of increased NET is tyrosine hydroxylase-positive neurons which have been shown to increase in the striatum after dopamine-depletion (Darmopil et al., 2008; Ünal et al., 2015). These neurons can be spiny projection neurons (Darmopil et al., 2008) or striatal interneurons (Ünal et al., 2015), which are considered to appear after a compensatory phenotypic conversion of a population of pre-existing striatal neurons. However, to our knowledge there have been no evidence of NET expressions in these neurons.

Conversely, there is one study in the striatum suggesting that NETs and norepinephrinergic neurons may not be significantly involved in reuptake of dopamine, even when dopaminergic neurons are significantly reduced. Navailles et al. (2014) showed in 6-OHDA-lesioned rats that the NET blockers desipramine (10 mg/kg) and reboxetine (3 mg/kg) potentiated the levodopa effect in the prefrontal cortex, substantia nigra pars reticulata, and hippocampus, but not in the striatum. They discussed that the contradictory result of Arai et al. (2008) is because of their high dose of desipramine (25 mg/kg), in which desipramine non-selectively binds to sites other than NET. However, Chotibut et al. (2014) used a lower dose of desipramine (12 mg/kg) and demonstrated a significant increase of levodopa-derived dopamine concentration in the dopamine-denervated striatum. The inconsistencies between studies may be due to residual DAT expression and activity of SERT and other monoamine transporters in the particular experimental situation (Figure 2B). Although it is difficult to interpret the discrepancies among studies, it remains possible that NET plays a role in reuptake of extracellular dopamine in the dopamine-denervated striatum.

## The role of low affinity transporters in the striatum: organic cation transporter-3 (OCT-3) and plasma membrane monoamine transporter (PMAT)

DAT, NET, and SERT are high-affinity transporters for dopamine, norepinephrine, and serotonin, respectively, all of which belong to the solute carrier (SLC) 6A family. They are the primary targets for drugs used to treat depression and other psychiatric disorders and may transport some neurotoxins in patients with drug addiction and animal models of the diseases (Giros and Caron, 1993; Torres et al., 2003). Other than these high-affinity amine transporters, additional transporters for biogenic amines in the brain have been identified, such as OCT (Gründemann et al., 1998; Eisenhofer, 2001; Vialou et al., 2004; Amphoux et al., 2006; Koepsell et al., 2007) and PMAT (Engel et al., 2004; Engel and Wang, 2005), which have low affinity but high capacity for monoamine transport. These low affinity transporters could become more important in the clearance of monoamines when high-affinity transporters do not work normally (Hensler et al., 2013), such as in PD when dopaminergic neurons and DAT are significantly reduced, and levodopa-derived dopamine is excessively released in the extracellular space.

### OCT-3

OCT is a member of the SLC superfamily, the organic cation/anion/zwitterion (SLC 22) family of transporters. Decynium-22 (D-22) can block OCT. OCT-1 and OCT-2 are mainly expressed in the liver and kidney, respectively (Lee et al., 2009). OCT-3 is expressed in various organs including the brain (Lee et al., 2009). In the central nervous system, OCT-3 is detected in glial cells and neurons in rodents (Vialou et al., 2004; Cui et al., 2009). Astrocytes in substantia nigra and striatum adjacent to soma and terminals of midbrain dopaminergic neurons express OCT-3, whereas those in cerebellum, hippocampus and cortex do not express OCT-3 (Cui et al., 2009). OCT-3 expression has also been reported in cultured human astrocytes (Yoshikawa et al., 2013). It is not clear which type of neurons express OCT-3.

OCT-3 depletion in mice increases extracellular dopamine elevation after 1-methyl-4-phenyl-1,2,3,6-tetrahydropyridine (MPTP) treatment. The same mouse model also shows elevated extracellular dopamine concentration after high-dose methamphetamine treatment, but not after low-dose treatment (Cui et al., 2009). These results suggest that OCT-3 has a role in the removal of excess extracellular dopamine when dopamine concentration is high and has overcome the capacity of DAT. OCT-3 depletion also hampered the release of toxic organic cation 1-methyl-4-phenylpyridinium from astrocytes and protected against MPTP-induced dopaminergic neurodegeneration in mice (Cui et al., 2009). In 6-OHDA-lesioned parkinsonian rats, OCT-3 inhibitor D-22 increases extracellular dopamine concentrations (Sader-Mazbar et al., 2013), although D-22 also inhibits PMAT.

These studies suggest OCT-3 plays a role in the regulation of dopamine concentration in the parkinsonian striatum and could be a target for PD treatment.

### PMAT

PMAT is a member of the SLC 29, equilibrative nucleoside transporter family. PMAT is also one of the D-22 sensitive transporters, which can be blocked by D-22, similar to OCT-3. In mice, PMAT is expressed exclusively in neurons, not in astrocytes (Dahlin et al., 2007). It is still unclear which type of neurons expresses PMAT. One study showed that PMAT is distributed in various neuron subtypes throughout the brain, including cholinergic interneurons in the striatum (Vialou et al., 2007). However, another study demonstrated that PMAT is expressed in cultured human astrocytes (Yoshikawa et al., 2013). Thus, the localization of PMAT remains unclear.

As mentioned in the preceding subsection, Sader-Mazbar et al. (2013) demonstrated that in 6-OHDA-lesioned rats, D-22 treatment increases extracellular dopamine in the striatum. They emphasized the inhibition of OCT-3, but D-22 can also inhibit PMAT. The result might partly be due to PMAT inhibition leading to reduced reuptake of dopamine via PMAT.

## Closing remarks

We reviewed levodopa-dopamine metabolism and pathways in which extracellular dopamine is reuptaken in the dopamine-denervated striatum. Most of the studies addressed were performed using rodent models of PD induced with neurotoxin. Further studies are needed to expand the discussion in this review to higher species.

To date, various drugs that modify dopamine metabolism and uptake, especially monoamine transporter inhibitors, have been investigated for treatment of PD motor and non-motor symptoms. Since the recent comprehensive review by Huot et al. (2015), new studies have been published. For example, Conti et al. examined the effect of clomipramine, amitriptyline, and desipramine (Conti et al., 2016). We also recently showed the effect of duloxetine, an SNRI, on motor symptoms of PD (Nishijima et al., 2016). These studies suggest that all SSRI, SNRI, and other monoamine transporter inhibitors potentially influence dopamine metabolism and clearance in the striatum of advanced PD patients. Thus, when these drugs are administered to PD patients for depression, pain or other non-motor symptoms, it is important to keep in mind that the drug may modify dopamine metabolism and uptake, leading to changes in motor symptoms and motor complications in PD patients. Furthermore, low affinity monoamine transporters such as OCT-3 and PMAT may be new targets to modify dopamine metabolism for PD treatment in future studies.

## Authors contributions

HN: reviewing the concept, compiling sources, interpreting the data, and writing the manuscript. MT: reviewing the concept, compiling sources, reviewing and critiquing the manuscript.

### Conflict of interest statement

The authors declare that the research was conducted in the absence of any commercial or financial relationships that could be construed as a potential conflict of interest.

## Abbreviations

3-MT: 3-methoxytyramine
3-OMD: 3-O-methyldopa
6-OHDA: 6-hydroxydopamine
AADC: aromatic amino acid decarboxylase
ALD.DH: aldehyde dehydrogenase
COMT: catechol-O-methyltransferase
D-22: decynium-22
D2R: dopamine D2 receptor
DA: dopamine
DAT: dopamine transporter
DCI: dopa-decarboxylase inhibitor
DOPAC: dihydroxyphenylacetic acid
DOPALD: dihydroxyphenylacetaldehyde
DR: dopamine receptor
HVA: homovanillic acid
MAO: monoamine oxidase
MPTP: 1-methyl-4-phenyl-1,2,3,6-tetrahydropyridine
NET: norepinephrine transporter, noradrenaline transporter
OCT: organic cation transporter
PD: Parkinson's disease
PMAT: plasma membrane monoamine transporter
SERT: serotonin transporter
SLC: solute carrier
SNRI: serotonin norepinephrine reuptake inhibitor
SSRI: selective serotonin reuptake inhibitor
VMAT-2: vesicular monoamine transporter-2.
